# Renal injury in obese children

**DOI:** 10.1080/0886022X.2019.1603111

**Published:** 2019-05-06

**Authors:** Tomoyuki Kawada

**Affiliations:** Department of Hygiene and Public Health, Nippon Medical School, 1-1-5 Sendagi, Bunkyo-Ku, Tokyo, 113-8602, Japan

I have read with great interest the paper entitled ‘Are there any new reliable markers to detect renal injury in obese children?’ by Bostan Gayret et al. [1]. The authors examined the serum and urine levels of several biomarkers to determine the renal injury in children with special reference to obesity, and serum levels of cystatin C, urine neutrophil gelatinase-associated lipocalin (NGAL) and osteopontin (OPN) levels were significantly higher in obese groups. I have some concerns on this study with special reference to insignificant indicators.

Firstly, Goknar et al. reported that obese children had higher urinary N-acetyl-beta-d-glucosaminidase (NAG) and kidney injury molecule-1 (KIM-1) values against those of healthy controls [2], and concluded that these two indicators should be recommended as screening markers for detecting early renal damage in obese children. Bostan Gayret et al. also used urinary and serum KIM-1, which showed no significant difference between obese and non-obese children. Relating to renal biomarkers, Csernus et al. presented a significant association between childhood obesity and glomerular/tubular protein excretion [3]. Namely, obese children had a higher degree of urinary albumin and beta-2-microglobulin (BMG) against those of healthy controls. Urinary albumin excretion (micro-albuminuria) is an indicator of disturbed glomerular permeability, and BMG is freely filtered through the glomerulus and almost completely reabsorbed by the renal proximal tubules. Namely, urinary BMG excretion would be a sensitive marker of proximal tubular dysfunction. In contrast, NAG cannot pass through the glomerulus, and urinary NAG is presented by the damage of renal proximal tubules. I recommend Bostan Gayret et al. including urinary BMG and NAG as biomarkers of renal injury in obese children to speculate the mechanism of renal damage by obesity.

Secondly, Han et al. [4] reported the reference values of urinary KIM-1 and matrix metalloproteinase-9 (MMP-9) in control children, which showed skewed distribution. This means that geometric transformation is more appropriate for the analysis. Bostan Gayret et al. presented both mean and median values of each biomarker in their Table 3, and I recommend the authors selecting statistical procedure, Mann–Whitney *U*-test or *t*-test, for analyzing the difference in each biomarker.

Finally, I previously conducted a cross-sectional study to know the association between urinary cadmium and blood pressure of adult inhabitants in a low-level of cadmium-polluted area [5]. There was a significant negative relationship between body mass index and the logarithm of creatinine-adjusted urinary NAG in male (*r*=－0.191, *p* < 0.05, *n* = 113). This significant relationship disappeared in female inhabitants. As a final concern, sex difference on the relationship should be verified in cases of children. In addition, the pathophysiology of the effect of obesity on renal injury in children has not been clearly described, and obesity-related renal damage should be verified by further studies.
